# Evaluation of an Ecological Momentary Intervention for Depression in Older Adults Living Alone

**DOI:** 10.1093/geroni/igab046.1671

**Published:** 2021-12-17

**Authors:** Heejung Kim, Soyun Hong, Sangeun Lee, Kijun Song, Mijung Kim, Yuntae Kim, Hyein Kim

**Affiliations:** 1 Yonsei University, Seoul, Seoul-t'ukpyolsi, Republic of Korea; 2 Yonsei university, Seoul, Seoul-t'ukpyolsi, Republic of Korea; 3 University of Illinois, Chicago, Illinois, United States; 4 Mapo Senior Welfare Center, Seoul, Seoul-t'ukpyolsi, Republic of Korea

## Abstract

Depression is a common but treatable mental health problem among older adults. Daily management and monitoring is very important; thus, diverse approaches have been developed. The objective of this research was to develop and pilot-test a counseling-plus-mobile health (mHealth) intervention using ecological momentary intervention (EMI) to reduce daily depressive mood for older adults living alone. Of 64 older adults living alone in community settings, 44 completed mHealth training and EMI for 7 weeks between October 2018 and October 2019. Study participants were randomized into experimental and control groups. The intervention was based on the protocol developed for an mHealth program for older Korean adults. Participants wore an actiwatch that measured their depressed moods four times a day for 2 weeks. Depressive symptoms were measured using the Hamilton Depression Rating Scale (K-HDRS) and the Korean version of the Short Geriatric Depression Scale (SGDS-K). Sleep quality was assessed using the Korean version of the Pittsburgh Sleep Quality Index (PSQI-K). The mean age of the study sample was 76.05±5.66 years, and the majority of the participants were female (61.0%, 36/59). There were no demographic characteristic differences between intervention and control groups. Based on multi-level modeling, EMI was not associated with significant improvements in depressive symptoms. However, depressive symptoms showed an initial decreasing trend, leveling off toward the end of the intervention period. This study finding could function as preliminary data to develop mHealth-based EMIs for older users in a larger, long-term study.

